# A Sensory Material Approach for Reducing Variability in Additively Manufactured Metal Parts

**DOI:** 10.1038/s41598-017-03499-x

**Published:** 2017-06-15

**Authors:** B. E. Franco, J. Ma, B. Loveall, G. A. Tapia, K. Karayagiz, J. Liu, A. Elwany, R. Arroyave, I. Karaman

**Affiliations:** 1 Department of Materials Science and Engineering, Texas A&M University, Texas, USA; 2 Department of Industrial and Systems Engineering, Texas A&M University, Texas, USA

## Abstract

Despite the recent growth in interest for metal additive manufacturing (AM) in the biomedical and aerospace industries, variability in the performance, composition, and microstructure of AM parts remains a major impediment to its widespread adoption. The underlying physical mechanisms, which cause variability, as well as the scale and nature of variability are not well understood, and current methods are ineffective at capturing these details. Here, a Nickel-Titanium alloy is used as a sensory material in order to quantitatively, and rather rapidly, observe compositional and/or microstructural variability in selective laser melting manufactured parts; thereby providing a means to evaluate the role of process parameters on the variability. We perform detailed microstructural investigations using transmission electron microscopy at various locations to reveal the origins of microstructural variability in this sensory material. This approach helped reveal how reducing the distance between adjacent laser scans below a critical value greatly reduces both the in-sample and sample-to-sample variability. Microstructural investigations revealed that when the laser scan distance is wide, there is an inhomogeneity in subgrain size, precipitate distribution, and dislocation density in the microstructure, responsible for the observed variability. These results provide an important first step towards understanding the nature of variability in additively manufactured parts.

## Introduction

Over the past decade, additive manufacturing (AM) has grown into a $5.1 billion industry with a 31.5% compound annual growth rate over 2012–2015^1^. A large portion of that growth is due to the rise of metal AM, which is currently being used for the production of customized implants for the biomedical^2^ and dental industries^3^, and very recently for the production of fuel nozzles for next-generation aircraft turbomachinery^4^. This growing interest is due to the ability of AM to produce complex near net-shape parts with high strength-to-weight ratios, computationally optimized geometries, and internal features, while reducing costs due to material waste and inventory storage^5^. In addition, recent studies have shown that these techniques can be used to create architectured materials with compositional gradients^6, 7^, property/microstructural gradients^8^, 4D functionality^9^, and/or location dependent properties^10^.

There remains, however, a serious concern regarding variability in the microstructure and mechanical properties of parts built by metal AM techniques. These parts can exhibit high degrees of in-sample variability (e.g. differences in functional response relative to the location in a single sample) as well as sample-to-sample variability^11^. This is a major obstacle against the implementation of AM in industries with stringent quality and part certification requirements such as the aerospace and biomedical sectors. Currently, the practice for addressing the high variability in meal AM revolves around conducting extensive experimentation to build statistically substantiated databases of the mechanical response^12^. Such approaches to variability characterization and control are highly specific to the material composition, the process technique, and even the manufacturer of the AM system. If any of these factors is changed, a new set of experiments must be conducted. Another approach involves investigating the microstructure and geometry of single laser printed tracks^13, 14^; however, this method does not provide sufficient insight into the complex microstructures and thermal histories that result from the interaction between multiple successive laser tracks. Instead, a more practical and flexible approach would be to predict the variability using computational tools based on modeling of the underlying physical phenomena,informed by quantitative measurements of the microstructural and compositional variability. However, such computational tools do not currently exist since the sources of variability and the physical mechanisms or processing parameters that control the variability are often not known in AM parts.

In addition, characterizing and understanding the sources of microstructural and compositional variability are problematic. Quantification of the variability must be performed on the macro-, micro-, and nano-scales, each of which requires the use of dedicated techniques. Characterizing nanoscale defects such as dislocations, inclusions, and precipitates is, in particular, a time consuming process, and it is difficult to generate quantifiable metrics in order to compare multiple samples or to observe the effect of changes in the processing technique.

Variability in metal AM parts originates from multiple sources. An important source of variability is the complexity of the underlying physical phenomena occurring during fabrication; these include large temperature gradients, extremely rapid solidification, and complicated thermal histories. Equally important source is the large number of manufacturing process parameters whose influence on the resulting microstructure and mechanical properties is not fully understood.

**Figure 1:**
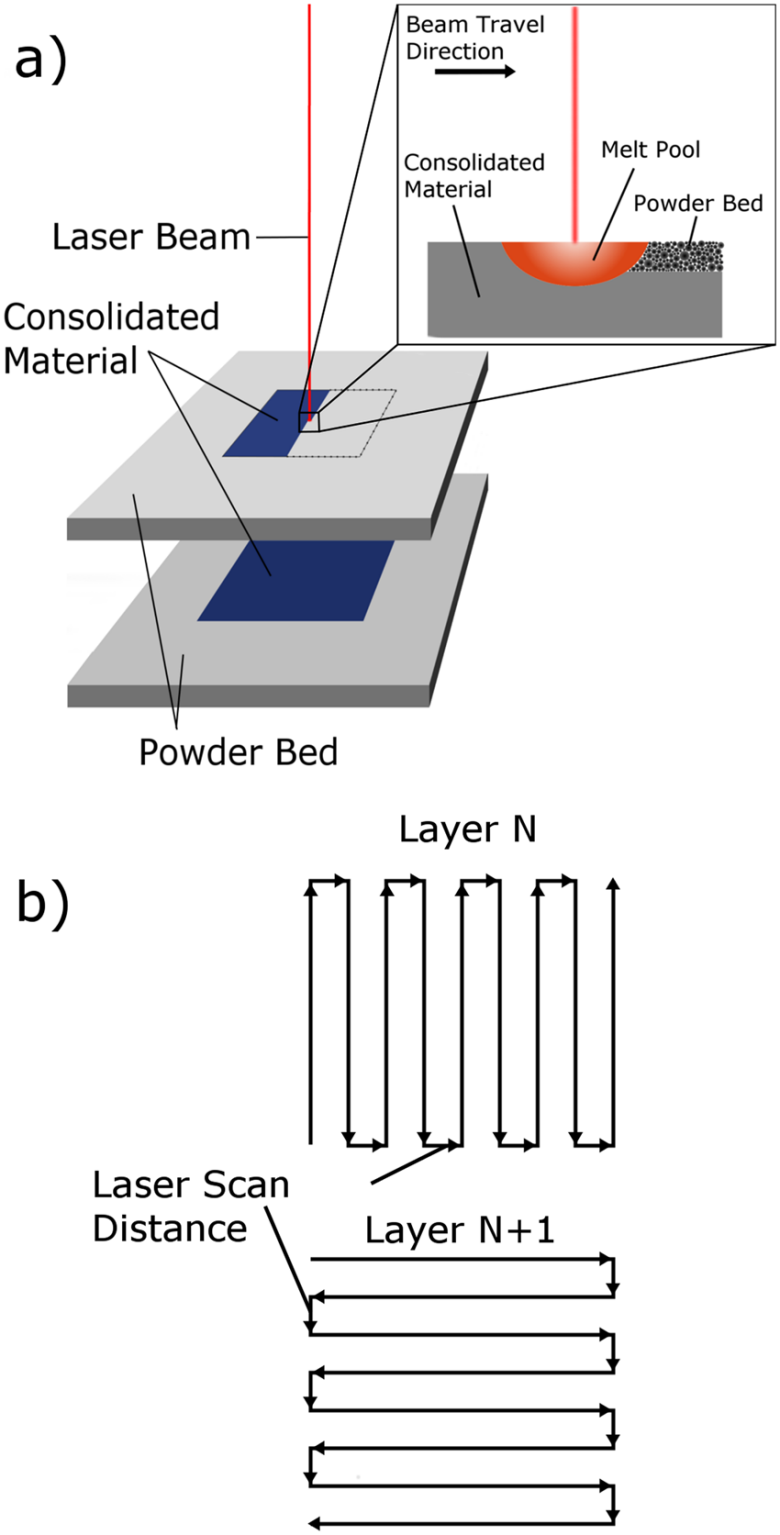
Schematic illustrations of the Selective Laser Melting (SLM) process, (**a**) showing the consolidation of a fully solid part in a layer-by-layer manner, and the consolidation of powder at the melt pool. The schematic in (**b**) shows the pattern of the laser as it rasters in a hatch pattern. The long axis of the hatches is rotated by 90° with every new layer.

In the present study, we use a novel approach in order to detect and reduce the in-sample and sample-to-sample variability in materials fabricated using Selective Laser Melting (SLM). SLM is a well-known powder bed based layer-by-layer technique that has been successfully used to consolidate a wide variety of metallic powders such as magnesium^15^, aluminum alloys^16, 17^, steels^18–20^, titanium alloys^21, 22^, and nickel alloys^23, 24^, as well as metal-matrix composites^25, 26^ and metallic glasses^27, 28^. Figure 1 shows a schematic illustrating the typical SLM process. Bulk parts are constructed by spreading thin layers of metallic powder (typically on the order of 30–60 μm) which are then selectively consolidated by a laser beam. The beam power, beam speed, powder layer thickness, and the laser pattern can all be controlled and are generally chosen to minimize defects or maximize mechanical properties of interest. These process parameters can also be selected in order to influence the resultant microstructure, for example changing the grain morphology from columnar to equiaxed in binary NiTi^29^, refining the size and distribution of dendritic structures in 718 Inconel^30^, and controlling the grain texture in 316 L stainless steel^31^.

Here, we use NiTi alloy as a “*sensory material*” in order to observe how SLM process parameters affect the compositional and microstructural variability without the need for direct, cumbersome measurements. NiTi alloys are well known for the shape memory effect (SME), as a result of a solid-to-solid reversible phase transformation, in particular, the reversible martensitic transformation^32^. The reversible martensitic transformation is typically and easily detected using differential scanning calorimetry (DSC) as the peaks in heat flow during heating or cooling of the material can be directly related to the enthalpy loss or gain by the system as it undergoes the phase transformation. The transformation temperatures in NiTi are highly sensitive to Ni content^33^ in the Ni-rich side of the stoichiometry, as well as the precipitate structure^34–36^, and grain size^37, 38^. Small differences in composition and microstructure therefore result in large differences in the transformation temperatures, which can be easily detected. This allows the detection of the effect of even small changes in process parameters on the resulting properties, therefore, the material acts as an “amplifier”. Although most materials used in additive manufacturing (such as Inconel, Ti6Al4V, and various steels) do not exhibit a reversible martensitic transformation, their mechanical properties are still a result of the microstructural changes that occur during the SLM process (e.g. changes that occur in grain morphology, precipitate distribution, composition due to evaporation, and dislocation density). These microstructural changes are similar to those that may be responsible for changes in the thermal transformation behavior in shape memory alloys.

By taking advantage of the sensory NiTi alloy and accompanying reversible martensitic transformation, we are able to observe that shortening the distance between adjacent laser scan lines (illustrated in Fig. 1b)—a process parameter among many others, also known as the “hatch distance”—below a critical value greatly reduces the in-sample and sample-to-sample variability in NiTi. To our knowledge this is the first report that clearly shows the link between process parameterization and variability in metallic AM parts, and these results can potentially be generalized to improve the mechanical response of other alloys prepared by AM methods.

## Results and Discussion

The material in the current work was a gas-atomized NiTi powder with an initial Ni content of 50.9 at%. Several sets of samples with various process parameters were fabricated but here only two sets of samples are reported which were fabricated using a constant laser power, laser speed, powder layer thickness, but with a distance between adjacent laser scans of 35 µm or 120 µm, which we refer to the “hatch distance”. Three different sample geometries were considered: 10 mm cubes, semicircles with a thickness of 1 mm and a diameter of 10 mm, and beams with a thickness of 1 mm and length of 60 mm. The fabricated samples were removed from the substrate using wire electrical discharge machining, after which DSC specimens were cut using the same method.

### Microstructural Analyses

**Figure 2:**
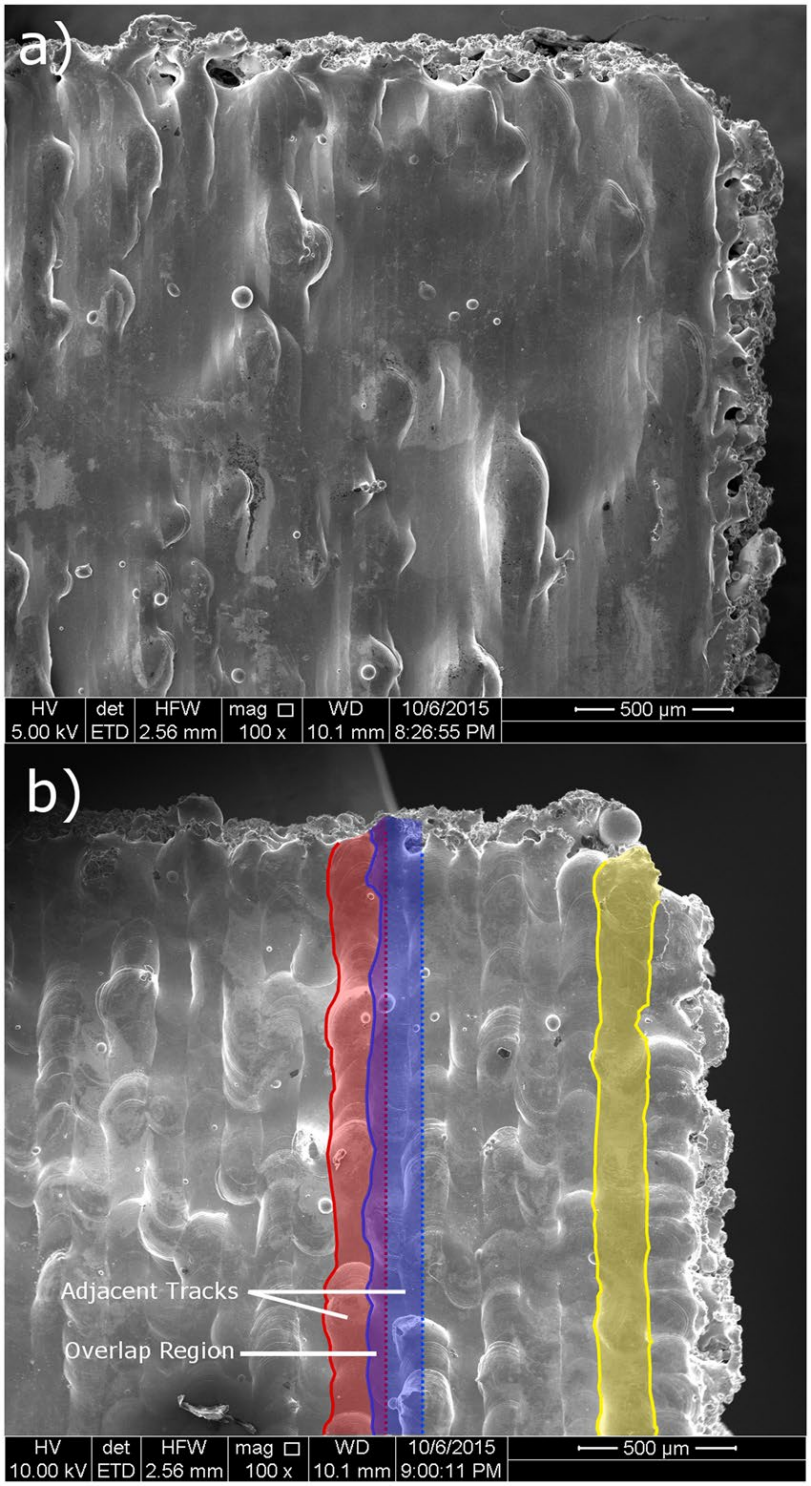
SEM images of the top surfaces of 3-D printed NiTi cubes using a distance between laser scans of (**a**) 35 μm and (**b**) 120 µm. The edges of the final laser track are highlighted on the right side of the image. In the center of the right image, two adjacent tracks are highlighted with their right edges estimated based on the width of the final track, showing the overlap region between them.

The SEM images in Fig. 2 show the as-printed top surfaces of the cubes with 35 µm and 120 µm hatch distances. The vertical striations visible in both images correspond to the path of the beam as it moves back and forth in a hatch pattern. In Fig. 2b, the final laser scan is highlighted to the right of the image, showing that the width of the melt pool is ~160 μm. Since the distance between adjacent laser scans is smaller than the width of the melt pool there is an overlap region where solidified material from the previous melting pass is re-melted. This is more easily seen in the 120 μm sample (Fig. 2b) where the right edge of each track is overlapped by the subsequent track. In the 35 μm samples, due to the smaller hatch distance, the powder at a given location is re-melted 4–5 times, while re-melting occurs only once in the areas where the tracks overlap in the 120 μm samples. Additionally, each time a new layer is consolidated, part of the previously solidified material underneath it is also re-melted. The powder layer thickness in the current study was 30 μm, and using a thermal model in COMSOL Multiphysics software, calibrated using pyrometer measurements, the depth of the melt pool is estimated to be 64 μm, which suggests that re-melting occurs in the two layers beneath the working surface.

### Detection of in-sample variability

**Figure 3:**
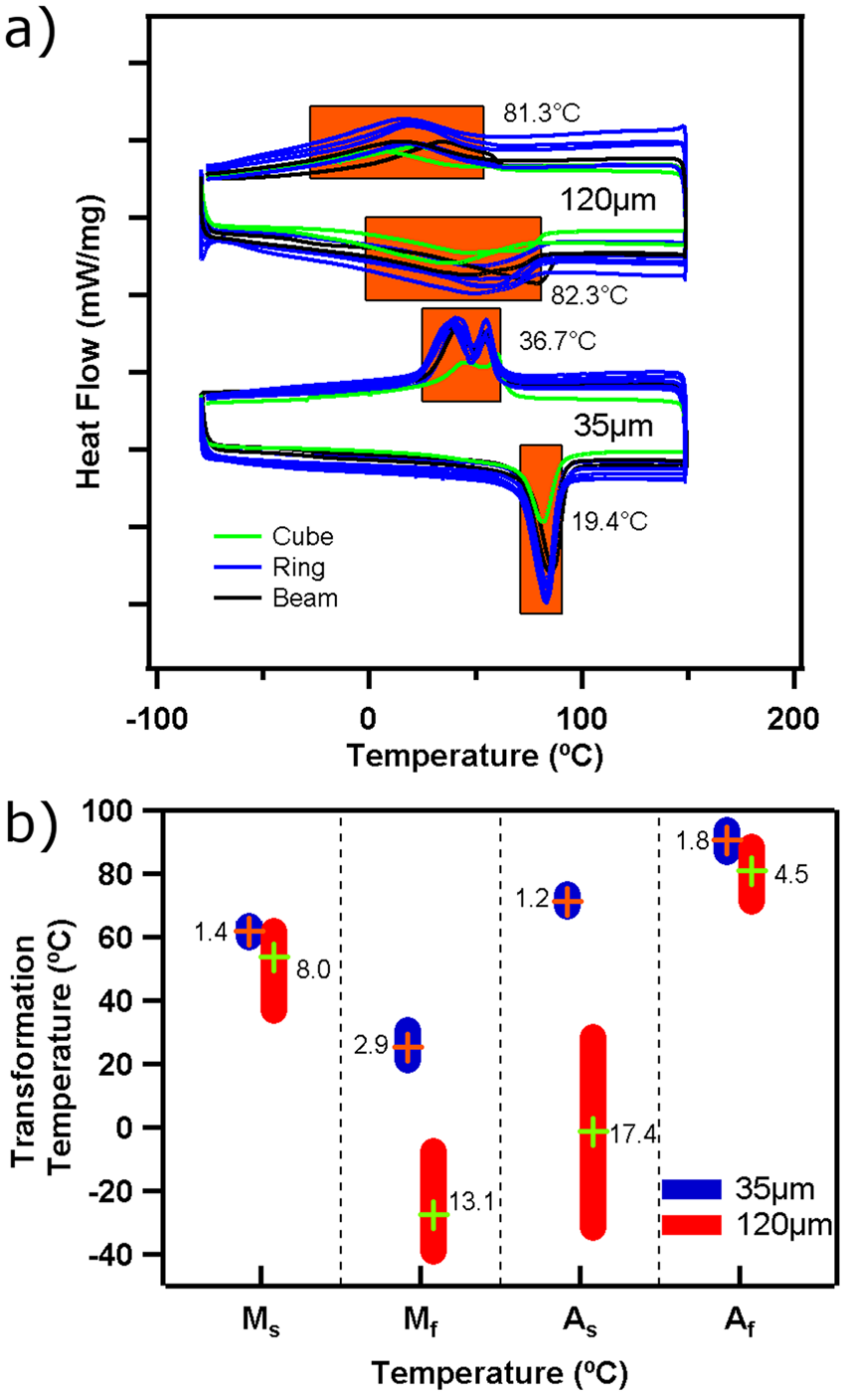
Results of DSC experiments for all NiTi samples 3-D printed using laser scan distances of 35 μm and 120 μm. The raw DSC curves are shown in (**a**) for multiple samples (10 repetitions with the same processing parameters). The transformation peaks are highlighted in orange, and the average width is indicated next to each peak. The graph in (**b**) shows the variability in measured transformation temperatures. The four columns correspond to the start (M_s_) and finish (M_f_) of the martensitic transformation during cooling, and the start (A_s_) and finish (A_f_) of the reverse transformation to austenite during heating. The height of the bars indicates the range of measurements, and the standard deviation is indicated beside each.

DSC experiments showed that in-sample variation was present in the 120 μm samples, and greatly reduced for the 35 μm samples. The results of the DSC experiments are shown in Fig. 3a. The in-sample variability is evident in the width of the peaks, which are more than double for the 120 μm samples compared to the 35 μm samples. In conventionally prepared Ni-rich NiTi, after homogenization by high temperature heat treatment, the peak width is around 10 °C^33^. The homogenization process creates a uniform microstructure and composition across all regions of the sample. This means that all parts of the material transform within a narrow temperature range, and the width of the resulting transformation peak is narrow. In the case of the SLM fabricated 120 μm samples the peak width is four times that of homogenized materials, and we attribute this to smooth microscale variations in composition and microstructure. Due to the large thermal gradient there is a large difference in thermal history across the melt pool. This can cause differences in precipitate volume fraction, precipitate size, matrix composition, and grain size, all of which alter the transformation characteristics. If the center of the melt pool reaches the boiling point, Ni will also vaporize at a higher rate than Ti, further changing the composition of the matrix^39^. The degree of vaporization, precipitate formation, grain size, and therefore the transformation temperatures will then depend on the distance from the center of the laser spot. A similar peak broadening phenomenon is observed in heat-treated NiTi with heterogeneous precipitation near grain boundaries^40^. In these cases, the transformation occurs over a very wide range with multiple distinct peaks that correspond to transformation in the grain interior and at the grain boundary^40, 41^. Compositional and microstructural differences also cause the peak widening in the 120 μm samples; however, the transformation peak is smooth because the heterogeneity in composition and microstructure is also smooth. In the case of the 35 μm samples the peak width is much narrower than those of the 120 μm samples because the thermal history across the material is more uniform. In the 120 μm samples there is a stark difference in thermal history at the center of the overlap zone and at the center of the laser tracks. In the 35 μm samples the shortened laser scan distance means that every location in the material is in an overlap zone, which leads to a more uniform thermal history. This uniform thermal history reduces the variation in composition and microstructure.

### Detection of sample-to-sample variation

Reducing the laser scan distance from 120 μm to 35 μm also reduces the sample-to-sample variation. This can be seen in Fig. 3b, which shows the range of DSC measured temperatures for the start and finish of the transformation during both heating and cooling. The numerical values can be found in the Supplementary Table S1. The standard deviation and range of measured values of the transformation temperatures for the 120 μm samples is several times larger than the 35 μm samples. We observe that the laser scan distance has a dramatic effect on the variability in the transformation temperatures from sample-to-sample; these transformation temperatures being M_s_ (austenite-to-martensite transformation start temperature), M_f_ (austenite-to-martensite transformation finish temperature), A_s_ (martensite-to-austenite transformation start temperature), and A_f_ (martensite-to-austenite transformation finish temperature). Reducing the laser scan distance from 120 µm to 35 µm reduces the M_s_ range from 25 °C to 5 °C, the M_f_ range from 32 °C to 9 °C, the A_s_ range from 60 °C to 4 °C, and the A_f_ range from 17 °C to 7 °C. We propose that the repeated melting that occurs when the laser scan distance is sufficiently narrow promotes homogenization of the inherent variation.

**Figure 4:**
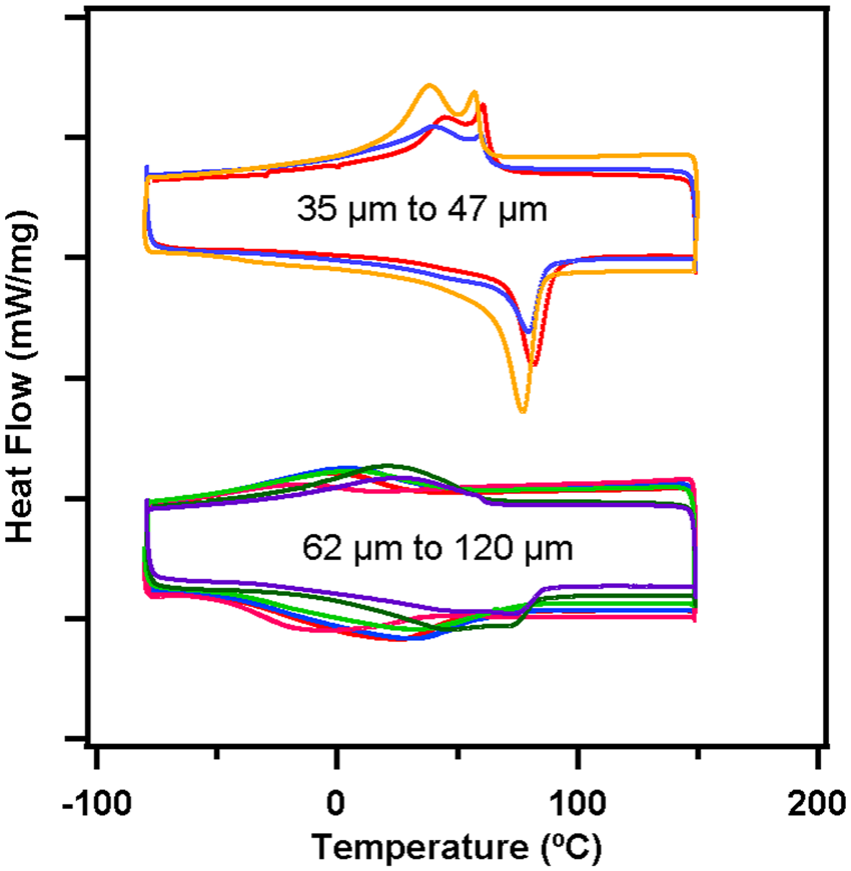
DSC curves for NiTi cubes 3-D printed with various process parameter values for laser power, speed, and scan distance. The curves are grouped by peak width.

DSC experiments were also performed on SLM fabricated cubes while varying parameters other than the laser scan distance (the laser power, scan speed, and scan distance). We observe that the in-sample variability seems to be more sensitive to laser scan distance than any other parameter. The relevant parameters and measurements are listed in Table S2. The DSC results in Fig. 4 show that samples printed with a laser scan distance over some critical value (which is in the range of 47–62 μm) exhibit wide transformation peaks, indicative of in-sample variability. The existence of this critical value is again related to the difference in thermal history at overlap zones and in the center of the laser tracks. The critical value represents a condition where the laser scans are sufficiently close enough so that the variation in thermal history, and therefore microstructural and compositional variation, is small.

### Microstructural origins of the property variations in AM fabricated NiTi

The effect of microstructural changes on the transformation temperatures in NiTi SMAs, fabricated using conventional ingot metallurgy techniques, has been extensively studied. In summary, the following has to be kept in mind to better understand the microstructural origins of the variability in AM fabricated NiTi:

- The transformation temperatures depend strongly on composition. When the Ni content is below 50 at.% the transformation temperatures remain constant. As the Ni content increases from 50 to 51 at.%, the transformation temperatures decrease by as much as 140 °C, and for compositions above 51 at.%, the transformation is completely suppressed^33^.
- Oriented (for example uniaxial) stresses have the effect of increasing the transformation temperatures. This relationship is generally linear, and in NiTi, the slope is around 6–10 MPa/°C^42^.
- Higher dislocation densities in NiTi result in broader transformation peaks^43^.
- Reduction in grain size down to the nanoscale (below 100 nm) suppresses the transformation. The transformation can be completely suppressed when the grain size is smaller than 60 nm^44^. However, if the grain size is in the microscale, then there is no effect on the transformation behavior.
- The formation of nanoprecipitates has dual effects. Precipitation of Ni-rich Ni_4_Ti_3_ and Ti-rich Ti_2_Ni changes the composition of the overall matrix, which affects the transformation temperatures. Additionally, in cases where the average distance between precipitates is very small (in the nanoscale) the transformation temperatures are suppressed^34^.

The differences in transformation behavior between the 35 μm and 120 μm samples can be understood to be primarily a result of differences in these microstructural effects. The 35 μm sample has narrow transformation peaks, indicating that material transforms at more or less the same temperature. This implies that the microstructure is homogenous. The broad peaks in the 120 μm indicate that regions of the sample transform at different temperatures; thus we can expect to observe local variations in microstructure. The effect of macro- and micro- scale residual stresses is negligible due to two reasons; after cutting the specimens free from the substrate, and then cutting the DSC samples from the specimen most of the residual stress has been relieved^45^. Additionally, according to the simulations by Gu and He, the residual stresses in SLM fabricated NiTi reach around 100 MPa after several layers have been formed^46^. This corresponds to a transformation temperature increase of only 10 °C, so residual stress can be ruled out as a factor in the present case. We therefore focus our investigation on micro- and nano-scale differences in composition, grain size, dislocation density, and precipitate distribution to understand the microstructural origins of the property variability in AM fabricated NiTi sensory material.

### Microscale Microstructural Characterization

**Figure 5:**
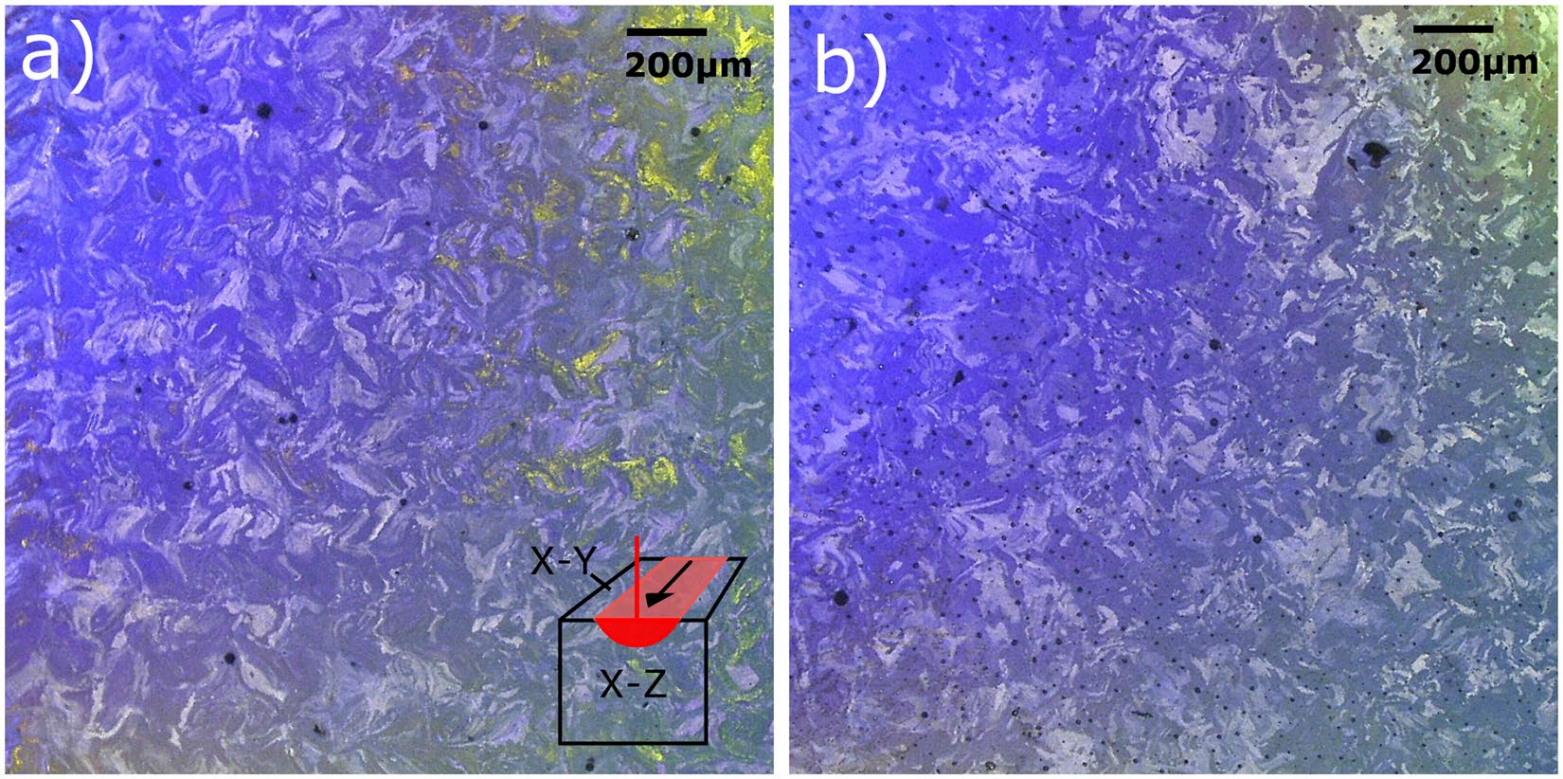
Optical microscopy images of the cross sections cut in the X-Y plane from the NiTi samples 3-D printed using laser scan distances of (**a**) 35 μm and the (**b**) 120 μm. The color contrast indicates differences in grain orientation. The small black spots in (**b**) are not porosity, but artifacts from the etching process.

Figure 5 shows the grain size and morphology for the 35 μm and 120 μm specimens in the X-Y (perpendicular to beam) plane. The grain morphology for the 35 μm sample shows the characteristic checkerboard pattern presented by Bormann *et al*.^29^, while the grains in the 120 μm sample appears to be more randomly structured. In the X-Z plane (shown in Figure S2) the columnar structure of the grains can be seen in both samples. The overall grain size for both specimens is on the order of one hundred microns, and other than the checkerboard pattern, there is no significant difference in grain morphology between the two specimens.

**Figure 6:**
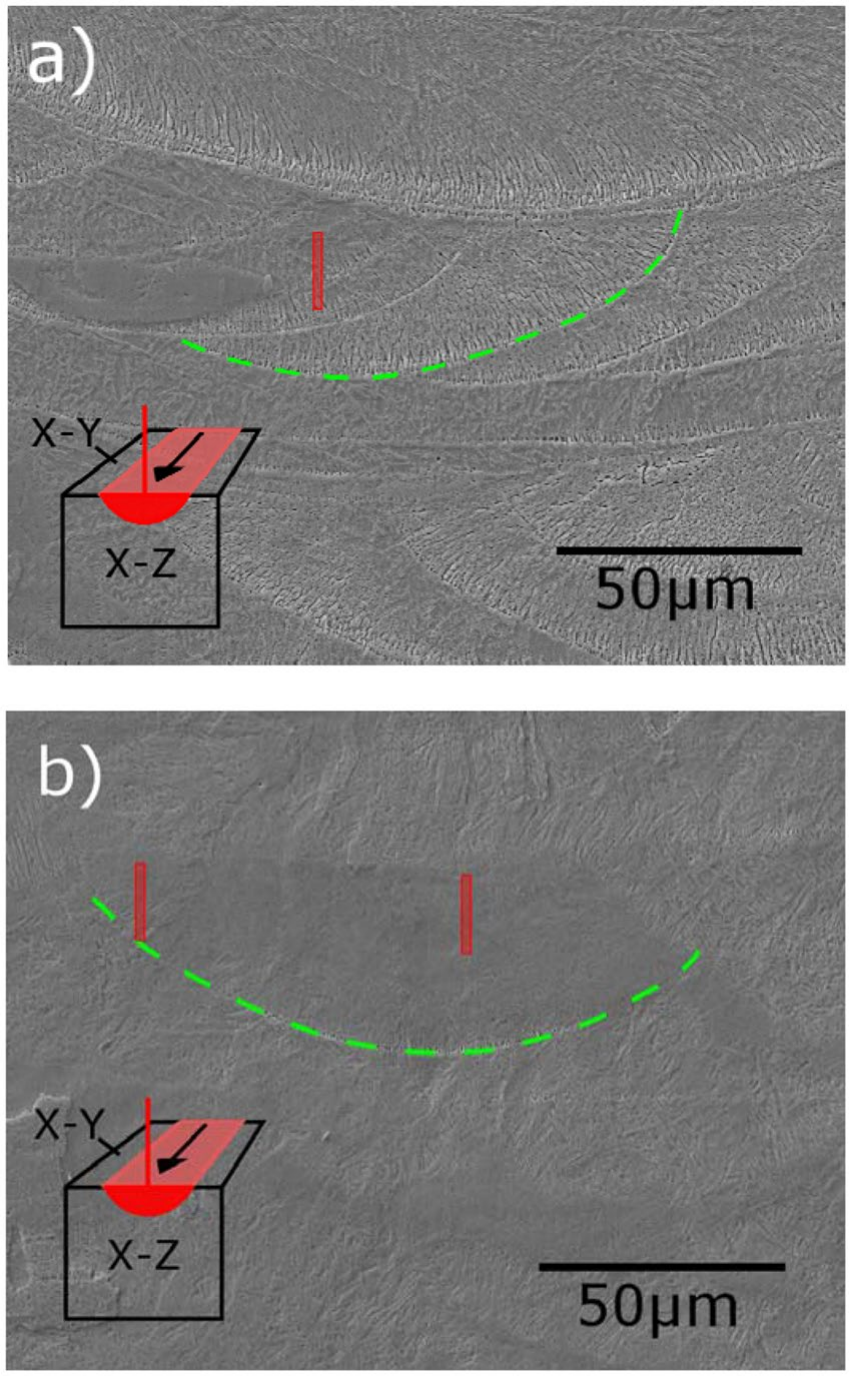
SEM images of the X-Z surfaces etched to reveal the cross sections of the NiTi samples 3-D printed using laser scan distances of (**a**) 35 μm and the (**b**) 120 μm. The visible portions of the laser tracks under investigation have been highlighted in green. The location of Focused Ion Beam (FIB)-prepared TEM specimens is highlighted in red.

To investigate compositional differences at the microscale, the specimens were prepared in the X-Z plane, revealing the cross sections of the individual laser tracks. SEM images of these cross sections are shown in Fig. 6. Energy dispersive X-ray spectroscopy (EDS) area scans and line scans (Supplemental Figure S1) in the region of the laser track cross sections revealed no significant compositional differences. This implies that if compositional differences do exist, they must be in the nanoscale in order to be undetectable by this method.

### Nanoscale Microstructural Characterization

Since no evidence for microscale grain and compositional differences was observed, we prepared transmission electron microscopy (TEM) specimens using the focused ion beam (FIB) technique in order to characterize the microstructure in the nanoscale at different locations within specific laser tracks. The locations where the TEM specimens were obtained from are highlighted in Fig. 6. For the 120 μm sample, TEM specimens were cut from the center and from the edge of the laser track, the center being in the single-melted region of the melt pool, and the edge being in the double-melted region where the track overlaps with the adjacent track. For the 35 μm sample, a single TEM specimen was cut from the center of the laser track which has also been affected by the melt pool of several adjacent tracks; this location has been remelted 4 times in the same layer.

**Figure 7:**
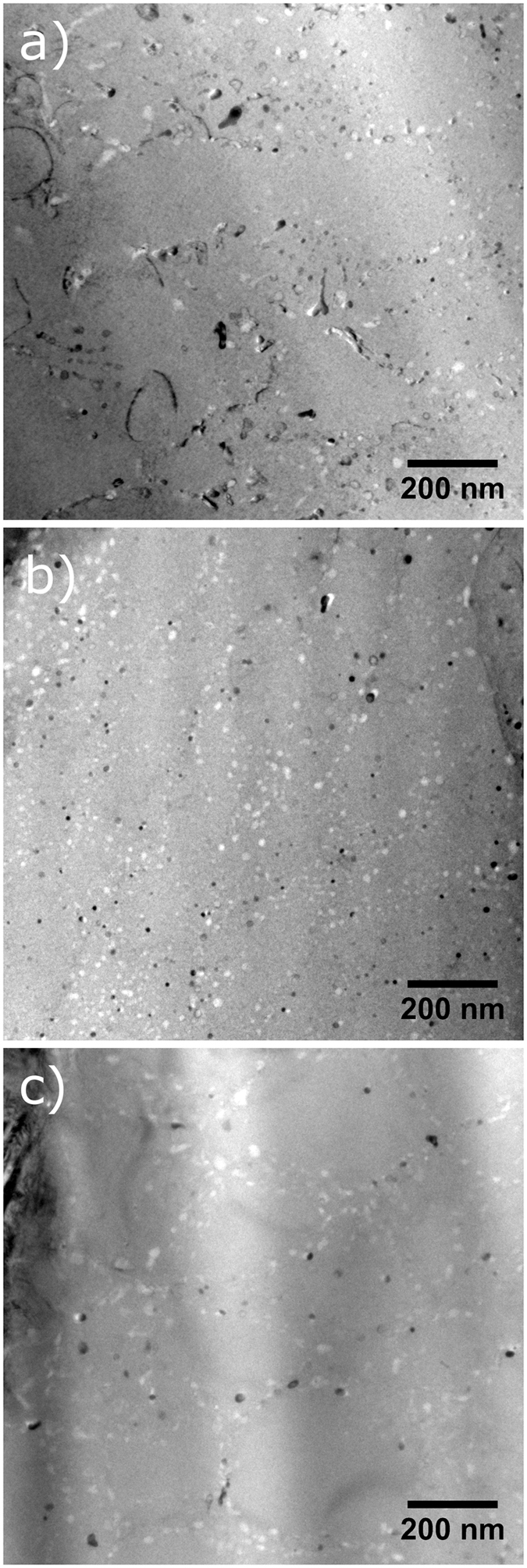
TEM images of the AM fabricated NiTi samples showing subgrain structure and precipitates decorating the subgrains in the (**a**) 35 µm hatch distance sample taken from the center of the laser track, the 120 µm hatch distance sample taken from (**b**) the center of the laser track, and (**c**) the edge of the laser track.

TEM imaging revealed differences in the microstructure between these three specimens. Figure 7 shows that inside each grain, there are large numbers of similarly oriented 200–400 nm subgrains, the boundaries of which are decorated with precipitates. The subgrain size is slightly larger in the 35 μm sample and at the 120 μm laser track edge sample in comparison to the 120 μm laser track center sample. Outlining the subgrains are 10–30 nm size precipitates: TEM-EDS compositional analysis (not shown) points to the composition of these precipitates as Ti_2_Ni. The distribution of the precipitates in the 35 μm and the 120 μm edge sample is mostly along subgrain boundaries; a slightly higher number density of precipitates inside of subgrain boundaries is observed for the 120 μm center sample.

**Figure 8:**
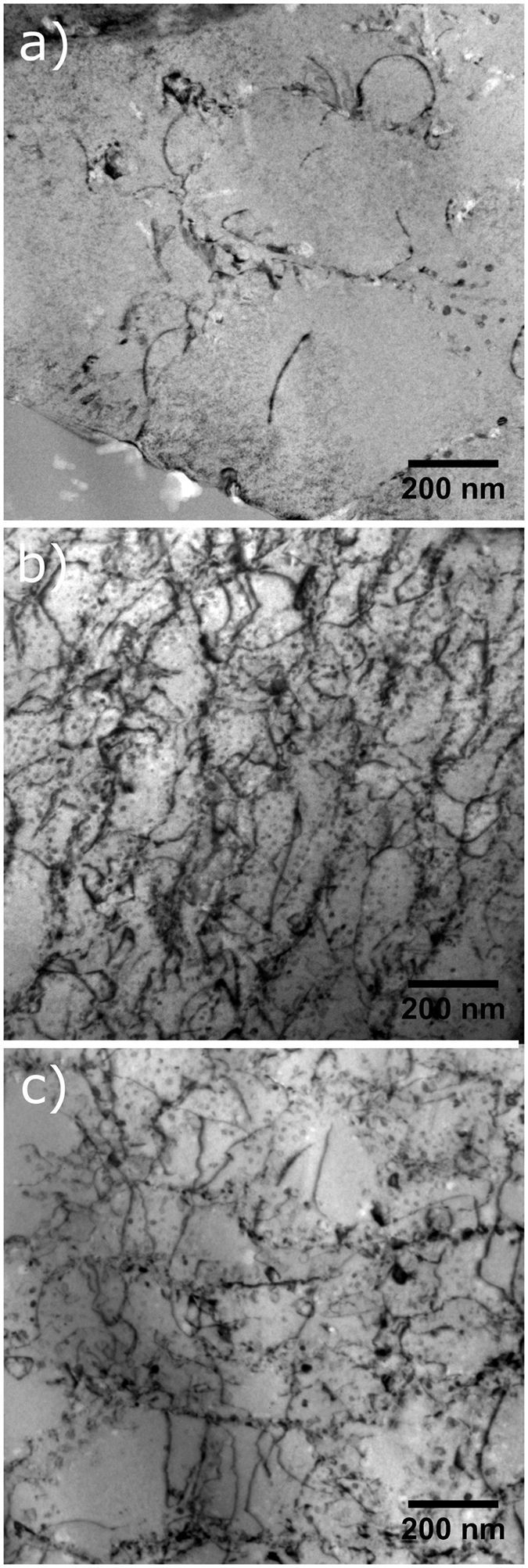
TEM images of the AM fabricated NiTi samples: (**a**) 35 µm hatch distance sample taken from the center of the laser track, the 120 µm hatch distance sample taken from (**b**) the center of the laser track, and (**c**) the edge of the laser track. Specimens have been tilted to show maximum contrast of the dislocations.

Figure 8 shows the TEM images of the same specimens tilted to reveal dislocations. The dislocation density is much lower in the 35 μm sample than in both of the 120 μm samples. The smaller hatch distance means that the material is subjected to a much higher number of reheating and remelting cycles. The dislocation density is also slightly lower for the 120 μm edge sample than in the center sample. Again, this is due to the remelting that occurs at the overlap zones of the laser tracks. This same mechanism must also be responsible for the differences in subgrain size and precipitate distribution in these samples.

**Figure 9:**
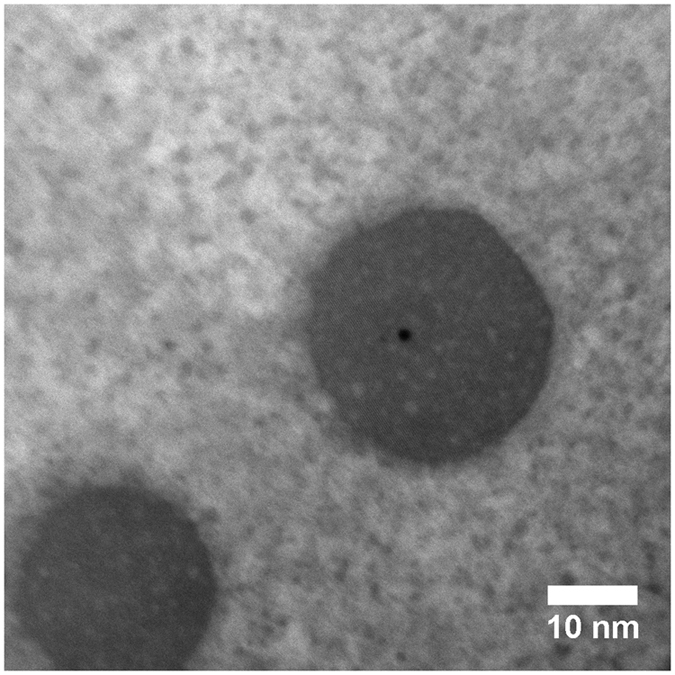
TEM image of the AM fabricated NiTi sample using laser scan distance of 35 μm showing small <5 nm precipitates along with two larger Ti_2_Ni precipitates.

Finally, we observe a second class of nanoprecipitates (Fig. 9) which are smaller than 5 nm. Imaging and compositional analysis of these precipitates was challenging, but they are present in large numbers throughout both the 35 μm and 120 μm samples. Since EDS of the matrix inside of the subgrains (without Ti_2_Ni particles) reveals that the composition is Ni-rich, these precipitates are most likely Ni-rich as well, possibly Ni_4_Ti_3_.

## Summary and Conclusions

In conclusion, we have shown that both the in-sample and sample-to-sample variabilities can be reduced in parts built via SLM by adjusting the process parameters (in this case, the laser scan distance, also called as hatch distance). There exists a critical distance value in the range of 47–62 μm above which localized microstructural and compositional variations have a visible effect on the transformation behavior. These microstructural variations were then captured by a variety of characterization methods, showing that when the hatch distance is wide, there is an inhomogeneity in subgrain size, precipitate distribution, and dislocation density. While the magnitude of the critical laser scan distance may vary from material to material, depending on the physical phenomena responsible for the microstructural heterogeneity and the sensitivity of material properties/performance metrics to the microstructural features, we believe that these results are applicable to a wide range of AM alloys systems. The results thus represent an important first step towards an understanding of the nature of the variability in AM parts.

## Methods

A commercial 50.9 at% NiTi rod with a 32 mm diameter was gas atomized to produce a powder with a d_50_ of 18.5 μm. The powder was consolidated using a 3D Systems ProX 100 3D printer using a laser power of 50 W, laser speed of 80 mm/s, and layer thickness of 30 μm. The samples were printed onto bulk NiTi substrates under a purged argon atmosphere. After printing, the samples were cut from the substrate using wire electrical discharge machining (EDM), which was then used to cut 2.5 × 2.5 × 1 mm DSC specimens. The DSC specimens were mechanically ground in order to remove the EDM contaminated surfaces.

A color chemical etchant method was used to reveal the grain structure under optical microscopy. The procedure followed that of Bormann^29^ and Escher^47^. EDM cut samples of the bulk specimens were initially mechanically polished, followed by electropolishing in an Electromet 4 with a 3 M H_2_SO_4_/alcohol solution. Finally, the slices were submerged in a solution of 15 ml HCL, 15 g Na_2_S_2_O_5_, 10 g K_2_S_2_O_5_, 2 g NH_4_Hf, and 120 ml deionized water. The images were captured in a Keyence VHX600 optical microscope with illumination and camera polarization adapters.

SEM observations were performed using a FEI Quanta 600 on the as-built surfaces of the cubes after ultrasonic cleaning in isopropyl alcohol. DSC experiments were performed in a TA instruments Q2000 under a flowing dry nitrogen atmosphere. The samples were sealed in aluminum pans, and heated and cooled at a rate of 10 °C/s.

TEM investigations were performed using a FEI Tecnai G2 F20 operating at 200 kV. TEM specimens were prepared from mechanically polished slices of the bulk SLM printed cubes using the FIB technique, with a gallium ion source Tescan Lyra-3 operating at 30 kV. TEM lamella with dimensions of 15 × 10 × 1 μm were cut using the standard lift-off method and welded to a copper grid holder, then thinned until transparent to low accelerating voltage (5 kV) secondary electron imaging.

## Electronic supplementary material

Supplementary Tables and Figures
