# Supplementary material for: A Sensory Material Approach for Reducing Variability in Additively Manufactured Metal Parts
Source: Sci Rep. 2017 Jun 15;7:3604. doi: 10.1038/s41598-017-03499-x (PMC5472568; doi:10.1038/s41598-017-03499-x)
Supplement: Supplementary file 1 — Supplementary Tables and Figures [file 41598_2017_3499_MOESM1_ESM.doc]

*Submitted to Scientific Reports in November 2016, Revised in April 2017*

**A Sensory Material Approach for Reducing Variability in Additively Manufactured Metal Parts**

B.E. Franco^a^, J. Ma^a^, B. Loveall^a^, G.A. Tapia^b^, K. Karayagiz^a^, J. Liu^a^, A. Elwany^b^, R. Arroyave^a^, I. Karaman^a^,*

*^a^Department of Materials Science and Engineering, Texas A&M University, USA*

*^b^Department of Industrial and Systems Engineering, Texas A&M University, USA*

*^*^ Correspondence and requests for materials should be addressed to I.K. (email: ikaraman@tamu.edu)*

**Supplementary Information.**

**Table S1.** Measured transformation temperatures for all 10 samples built using both 35µm and 120 µm laser scan distances.

| Sample | Laser Scan Distance  [µm] | M_s_  [°C] | M_f_  [°C] | A_s_  [°C] | A_f_  [°C] |
| --- | --- | --- | --- | --- | --- |
| Cube | 35 µm | 62 | 27 | 72 | 91 |
| Semicircle | 35 µm | 62 | 22 | 70 | 90 |
| Semicircle | 35 µm | 62 | 21 | 71 | 92 |
| Semicircle | 35 µm | 62 | 21 | 71 | 92 |
| Semicircle | 35 µm | 62 | 25 | 73 | 90 |
| Semicircle | 35 µm | 62 | 25 | 72 | 92 |
| Beam | 35 µm | 60 | 23 | 70 | 91 |
| Beam | 35 µm | 61 | 26 | 74 | 94 |
| Beam | 35 µm | 63 | 31 | 72 | 92 |
| Beam | 35 µm | 62 | 26 | 70 | 87 |
| Cube | 120 µm | 61 | -33 | -6 | 81 |
| Semicircle | 120 µm | 60 | -35 | -11 | 85 |
| Semicircle | 120 µm | 57 | -32 | -4 | 81 |
| Semicircle | 120 µm | 57 | -9 | 28 | 78 |
| Semicircle | 120 µm | 52 | -31 | -4 | 82 |
| Semicircle | 120 µm | 55 | -8 | 4 | 80 |
| Beam | 120 µm | 62 | -10 | 24 | 88 |
| Beam | 120 µm | 37 | -39 | -32 | 71 |
| Beam | 120 µm | 55 | -39 | -1 | 81 |
| Beam | 120 µm | 43 | -39 | -13 | 82 |

**Table S2.** Process parameters for 10mm cube samples manufactured while varying the laser power, laser speed, and hatch distance.

| Laser Power [W] | Laser Speed [mm/s] | Laser Scan Distance [µm] |
| --- | --- | --- |
| 50 | 70 | 120 |
| 49 | 85 | 106 |
| 48 | 89 | 113 |
| 48.5 | 109 | 62 |
| 50 | 80 | 120 |
| 45.5 | 94 | 68 |
| 50 | 80 | 35 |
| 41 | 85 | 40 |
| 49 | 91 | 47 |


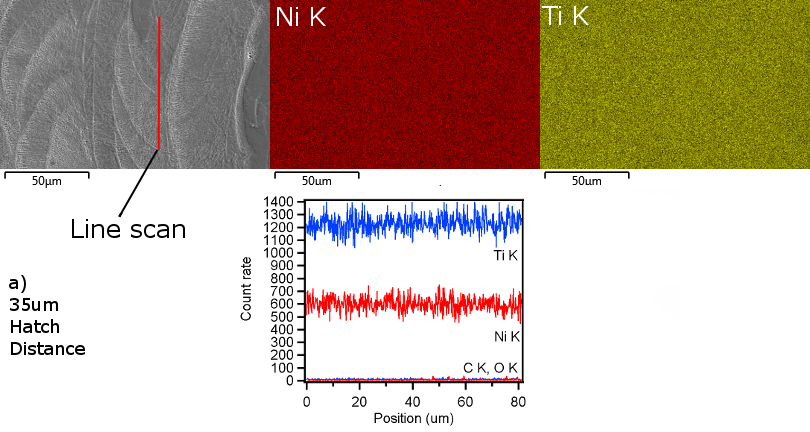


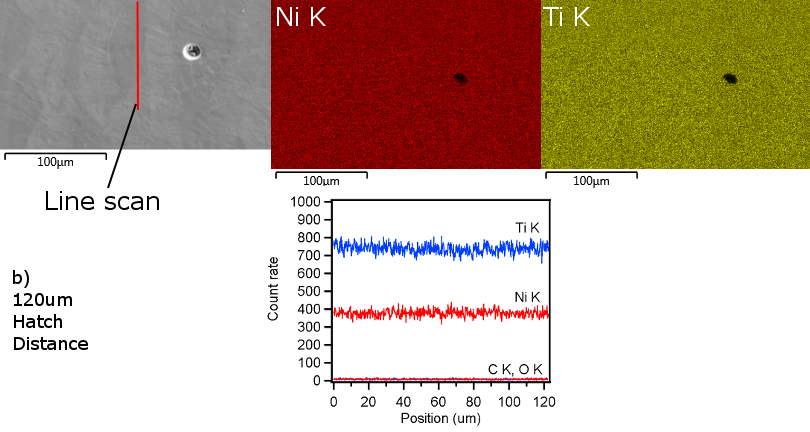


**Figure S1:** Energy dispersive X-ray spectroscopy (EDS) area maps and line scans of the NiTi samples 3-D printed using laser scan distances of (a) 35μm and the (b) 120μm. EDS experiments showed no significant compositional differences.


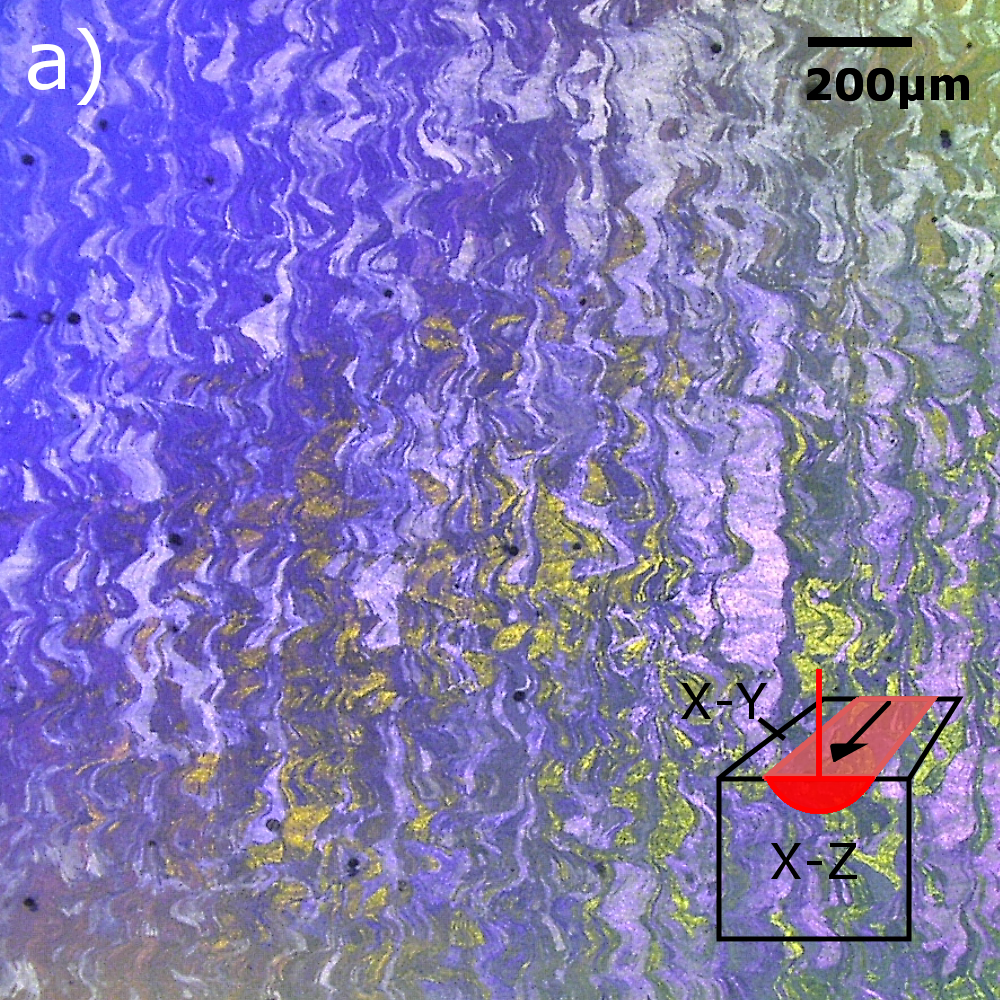

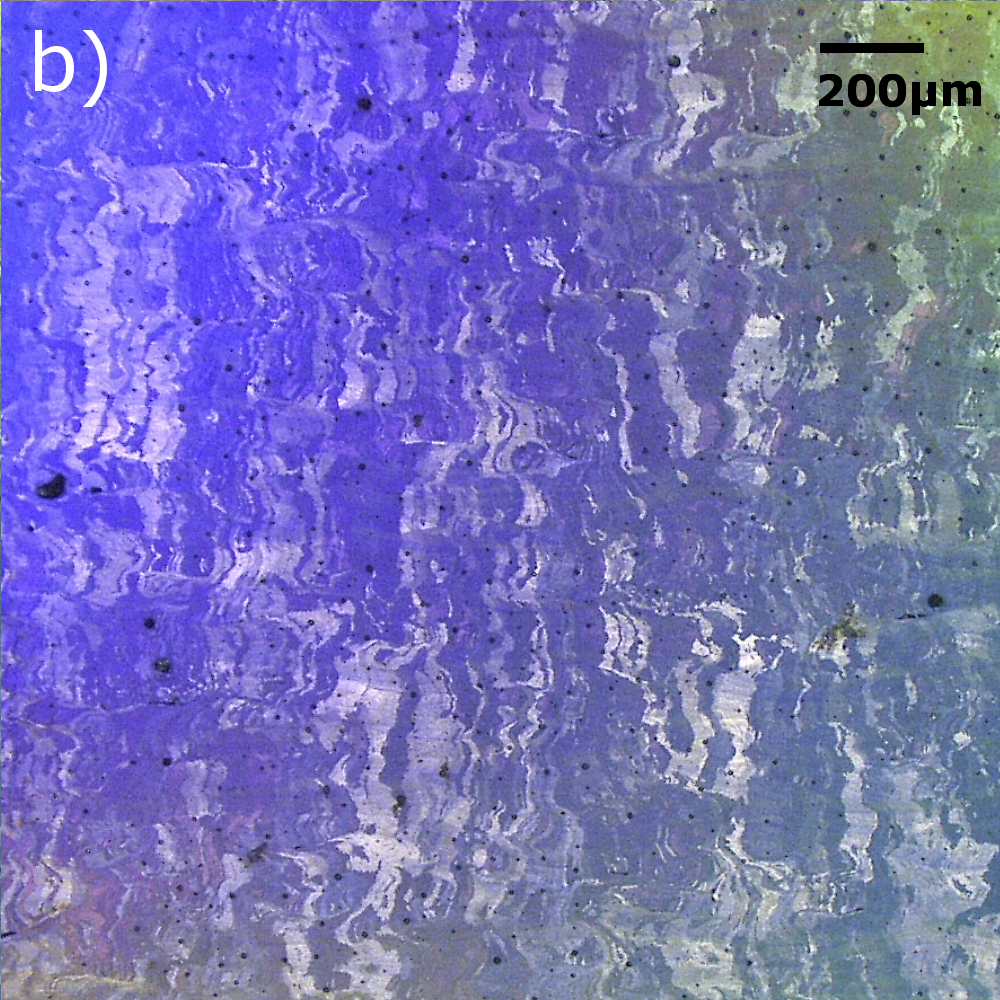


**Figure S2.** Optical microscopy images of the cross sections cut in the X-Z plane from the NiTi samples 3-D printed using laser scan distances of (a) 35μm and the (b) 120μm. The color contrast indicates differences in grain orientation.
